# WO_3_ Nanowire/Carbon Nanotube Interlayer as a Chemical Adsorption Mediator for High-Performance Lithium-Sulfur Batteries

**DOI:** 10.3390/molecules26020377

**Published:** 2021-01-13

**Authors:** Sang-Kyu Lee, Hun Kim, Sangin Bang, Seung-Taek Myung, Yang-Kook Sun

**Affiliations:** 1 Department of Energy Engineering, Hanyang University, Seoul 04763, Korea; chemiphile7@gmail.com (S.-K.L.); rlagns0829@nate.com (H.K.); b930403@gmail.com (S.B.); 2 Department of Nanotechnology and Advanced Materials Engineering & Sejong Battery Institute, Sejong University, Seoul 05006, Korea

**Keywords:** lithium-sulfur batteries, tungsten oxide nanowire, interlayer, thiosulfate mediator

## Abstract

We developed a new nanowire for enhancing the performance of lithium-sulfur batteries. In this study, we synthesized WO_3_ nanowires (WNWs) via a simple hydrothermal method. WNWs and one-dimensional materials are easily mixed with carbon nanotubes (CNTs) to form interlayers. The WNW interacts with lithium polysulfides through a thiosulfate mediator, retaining the lithium polysulfide near the cathode to increase the reaction kinetics. The lithium-sulfur cell achieves a very high initial discharge capacity of 1558 and 656 mAh g^−1^ at 0.1 and 3 C, respectively. Moreover, a cell with a high sulfur mass loading of 4.2 mg cm^−2^ still delivers a high capacity of 1136 mAh g^−1^ at a current density of 0.2 C and it showed a capacity of 939 mAh g^−1^ even after 100 cycles. The WNW/CNT interlayer maintains structural stability even after electrochemical testing. This excellent performance and structural stability are due to the chemical adsorption and catalytic effects of the thiosulfate mediator on WNW.

## 1. Introduction

Next-generation batteries have been in the spotlight to meet the demand for batteries with higher energy densities than commercial lithium-ion batteries [1,2,3,4,5,6]. Among them, lithium-sulfur batteries have been actively investigated owing to their attractive features: (1) ultrahigh theoretical energy density (2660 Wh kg^−1^), (2) worldwide abundant sulfur resources as low-cost active materials, and (3) lower environmental impact because their main constituents are sulfur and carbon [7,8,9,10].

Lithium-sulfur batteries are based on a general reaction mechanism; during discharge, high-order lithium polysulfides (Li_2_S_n_, when *n* ≥ 4) are generated and gradually converted into low-order lithium polysulfides (Li_2_S_n_, when *n* < 4) during lithiation (reduction) to finally form Li_2_S. Subsequent recharging drives delithiation (oxidation), so the cycle can be repeated [11]. High-order lithium polysulfides are soluble in the electrolyte, causing a shuttle phenomenon that induces the loss of active materials during the charging process and, consequently, incomplete battery charging [12,13]. By contrast, the use of volatile electrolytes exacerbates the electrolyte deficiency phenomenon, which results in lithium dendrite growth and deterioration in the stability of the cells [14]. In addition, sulfur (as active material) and lithium sulfides (as discharge products) are insulators that encourage the compositization of sulfur with electro-conducting materials [11]. For instance, porous carbons are capable of supporting large amounts of sulfur, and graphene and carbon nanotubes (CNTs) are proven applications in the fabrication of conductive carbon-sulfur composites [7,15]. Metal oxides, doped carbon, and modified membranes inhibit the migration of polysulfides to improve capacity retention during cycling [16,17].

Recent improvements in the performance of lithium-sulfur cells are attributed to film-type interlayers between the separator and the cathode. These interlayers facilitate electron transfer and prevent the migration of the produced lithium polysulfide toward the anode, thereby minimizing the loss of active materials to significantly improve capacity retention during cycling [16,17,18,19]. Inspired by metal–air batteries, metal oxides such as TiO_2_, MnO_2_, V_2_O_5_, and ZnO are of interest because of their ability to capture polysulfides as thiosulfate species [20,21,22,23,24]. More recently, Lin et al. introduced the catalytic effect of oxygen-deficient WO_3−δ_ (δ = 0.1) nanoplates on the reduction of polysulfides to Li_2_S_2_ or Li_2_S [25]. Choi et al. applied a ~100 nm-thick WO_3_ layer onto a graphene-sulfur composite, achieving capacity retention of 95% after 500 cycles [26]. Yang et al. demonstrated the ability of WO_3_ and carbon nanofibers, i.e., WO_3_-decorated N, S co-doped carbon nanofibers, to trap polysulfides and form an electro-conducting network [27]. These studies demonstrate the catalytic efficacy of WO_3_ in reducing high-order polysulfides to low-order Li_2_S.

It is anticipated that this catalytic effect of WO_3_ can be significantly enhanced by maximizing the active surface area through the formation of WO_3_ nanowires (WNW). Herein, we introduce a viable WNW/carbon nanotube (CNT) composite interlayer between the S electrode and separator. As discussed, WNW and lithium polysulfide interact to produce thiosulfate, which combines with high-order polysulfides to form low-order polysulfide and polythionate complexes. This mediating reaction enhances the kinetics of Li_2_S formation during discharge and S formation during charge. The loss of active material is mitigated by the facile conversion of S to Li_2_S, which suppresses the migration of high-order polysulfides toward the anode. Owing to the thiosulfate mediation on the surface of the WNW/CNT interlayer, the sulfur electrode exhibits excellent electrochemical capacity and rate capability even at high sulfur mass loadings of 4.2 mg cm^−2^.

## 2. Results and Discussion

**Figure 1:**
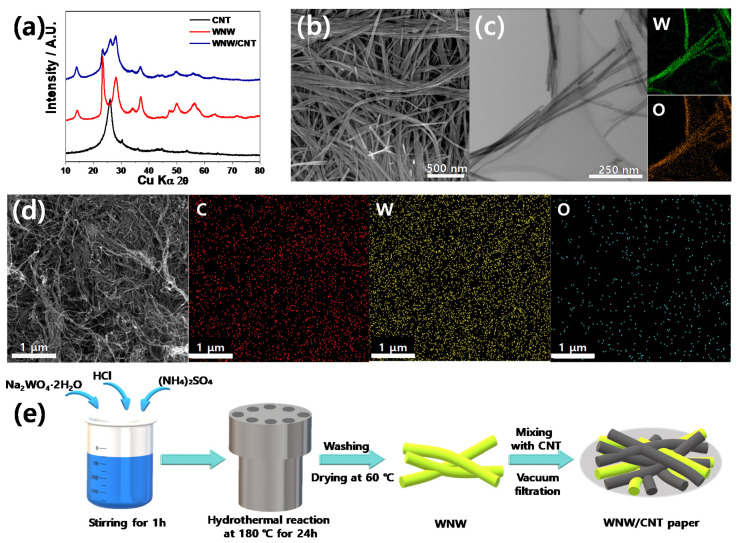
(**a**) XRD patterns of carbon nanotube (CNT), WO_3_ nanowires (WNW), and WNW/CNT. (**b**) SEM and (**c**) TEM image of WNW. (**d**) SEM images of WNW/CNT interlayer with EDS mapping images of carbon, tungsten, and oxygen. (**e**) Synthesis of the WNW/CNT interlayer.

During the fabrication of the WNW/CNT composite, a simple hydrothermal reaction occurred between Na_2_WO_4_∙2H_2_O and HCl; namely, Na_2_WO_4_∙2H_2_O + 2HCl → WO_3_ + 2NaCl + 3H_2_O (1), resulting in the formation of WO_3_ with a P6/mm space group (a = 7.298 Å and c = 3.899 Å) (Figure 1a). Images obtained through SEM and TEM indicate that the synthesized WO_3_ has a long nanowire shape with a thickness of approximately 20 nm (Figure 1b,c), which is similar to the morphology of CNTs (Figure S1a). Reaction (1) is likely responsible for the nucleation of WO_3_, while the subsequently added ammonium sulfate determines the one-directional growth of the WO_3_ particles. The homogeneous distribution of W and O on the nanowires is evident in the inset of Figure 1c [28]. The WNW has a high surface area of 111.31 m^2^ g^−1^ and a total pore volume of 0.48 cm^3^ g^−1^ according to nitrogen adsorption/desorption analysis (Figure S1b). The pore diameter distribution curve reveals that the size of the mesopores is centered at ~5.0 nm (Figure S1c). The produced WNWs were mixed with CNTs to fabricate WNW/CNT paper, which is prepared by the same method to fabricate the CNT paper (Figure 1d). The XRD patterns of the CNT and WNW/CNT composite reflect a CNT-derived peak at approximately 26° (2 θ) (Figure 1a). Despite the similarity in morphology between the nanowires, element maps show the presence of C, W, and O elements, indicating the successful fabrication of WNW/CNT paper, as summarized in Figure 1e.

**Figure 2:**
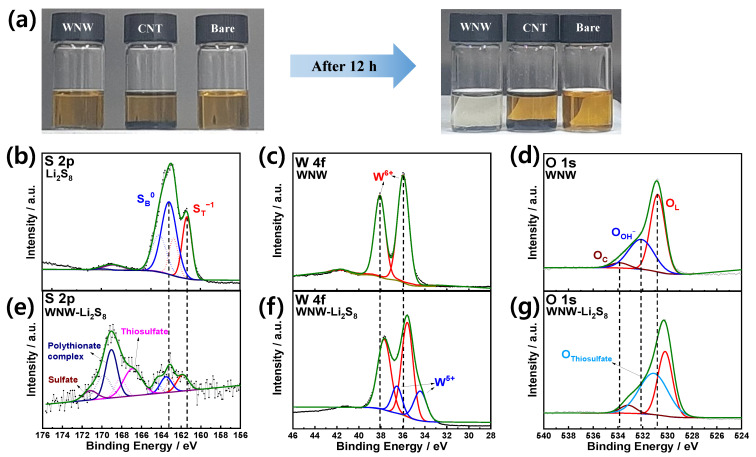
(**a**) Photographs of lithium polysulfide (0.0025 M Li_2_S_8_ 2 mL) electrolyte adsorption test. (**b**) XPS S 2p spectrum of Li_2_S_8_. XPS spectra of WNW: (**c**) W 4f and (**d**) O 1s. XPS spectra of WNW after Li_2_S_8_ adsorption test (**e**) S 2p, (**f**) W 4f, and (**g**) O 1s.

To confirm the thiosulfate mediation effect on the surface of WNW, a 2 mL lithium polysulfide solution (0.0025 M Li_2_S_8_ in DME/DOL) was added to vials containing 10 mg of CNT and WNW, respectively (Figure 2a). After aging for 12 h, the transparent yellow-colored WNW-Li_2_S_8_ solution turns colorless. In contrast, the transparent yellow-colored CNT-Li_2_S_8_ solution shows no color change after aging for 12 h, indicating that the lithium polysulfide (Li_2_S_8_) has not been adsorbed by the CNT.

**Figure 3:**
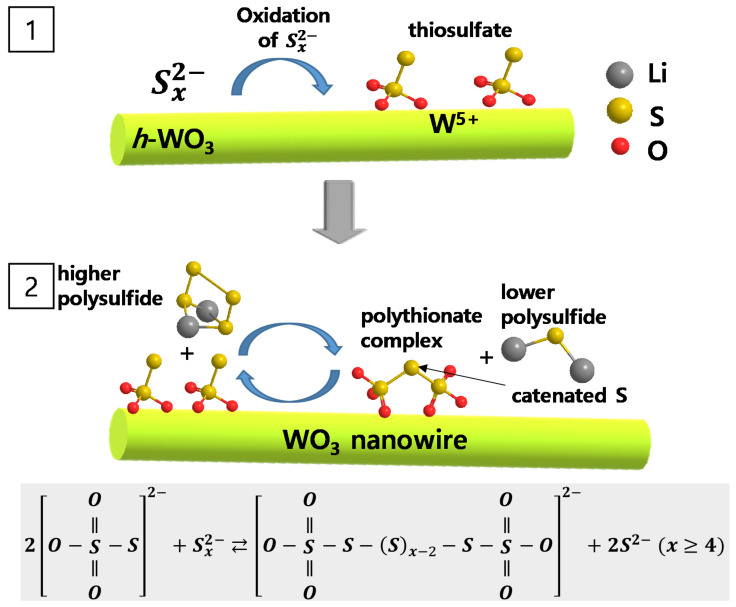
Schematic of lithium polysulfide conversion catalyzed on WO_3_ nanowire.

As shown in Figure S1b,c, the large specific surface area and pores of WNW maximize the reaction sites in contact with the lithium polysulfide. The chemical states of the surface elements of WNW in the aged WNW-Li_2_S_8_ solution were observed using XPS (Figure 2b–g). Li_2_S_8_ shows two S 2p_3/2_ peaks at 161.4 and 163.2 eV, assigned to the terminal (S_T_^−1^) and bridging sulfur (S_B_^0^) atoms, respectively (Figure 2b) [29]. WNW displays a pair of peaks at 36.0 and 38.1 eV, ascribed to W 4f_7/2_ and W 4f_5/2_, respectively, indicating that the synthesized WO_3_ nanowire is composed of W^6+^ (Figure 2c) [30]. The O 1s XPS spectrum shows a dominant peak at 530.8 eV that is attributed to the lattice oxygen of WNW, while the broad shoulder at 532.1 and 533.8 eV are due to contaminants such as hydroxyl groups or carbonates on the surface of WNW (Figure 2d) [31]. Figure 2e–g show the XPS spectra of the lithium polysulfide adsorbed on WNW (in the WNW-Li_2_S_8_ solution after aging for 12 h). While Figure 2b reflects dominant peaks corresponding to the terminal (161.4 eV) and bridging (163.2 eV) sulfur atoms in Li_2_S_8_, the S 2p spectrum of WNW-Li_2_S_8_ in Figure 2e shows dominant S 2p_3/2_ peaks at 166.6 and 169.0 eV. The S 2p_3/2_ peak at 166.6 eV corresponds to the thiosulfate binding energy, while the peak at 169.0 eV is ascribed to the polythionate complex [29]. Interestingly, in the W 4f spectrum of WNW-Li_2_S_8_, the binding energies of the two peaks corresponding to W^6+^ are 0.4 eV less than those in Figure 2c, and are accompanied by the emergence of peaks at 34.4 and 36.5 eV (Figure 2f). These peaks are attributed to W^5+^ as a result of the formation of WS_x_O_y_ oxysulfide species [32,33,34]; that is, the oxidation of high-order lithium polysulfide to thiosulfate is accompanied by the reduction of W^6+^ to W^5+^. The peaks of the O 1s spectrum of WNW-Li_2_S_8_ appear at lower binding energies than those of the O 1s spectrum of WNW, while the area of the center peak at 531.1 eV is greater than that of the corresponding peak in Figure 2d owing to the formation of thiosulfate. Based on the XPS data, it is reasonable to conclude that thiosulfate is produced, since polysulfides provide electrons to the WNWs at the interface, thereby reducing W^6+^ to W^5+^. The produced thiosulfate combines with high-order polysulfides to form polythionate complexes and low-order polysulfides. The thiosulfate formed on the WNW surface acts as a redox mediator and facilitates lithium polysulfide conversion. This process is summarized in Figure 3.

**Figure 4:**
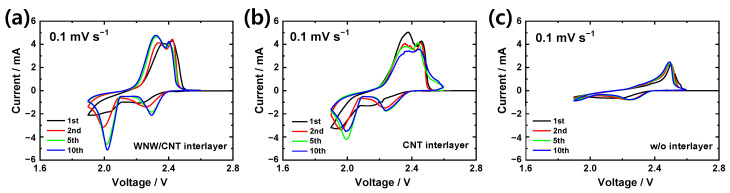
Cyclic voltammetry curves of lithium-sulfur cells with (**a**) WNW/CNT interlayer, (**b**) CNT interlayer, and (**c**) without an interlayer.

Cyclic voltammetry (CV) data demonstrate the improved reversibility of the sulfur electrode (S: 3 mg cm^−2^) in the presence of a WNW/CNT interlayer (Figure 4). For the cell containing a WNW/CNT interlayer (Figure 4a), a reduction peak is observed at 2.23 V during the first cathodic scan, associated with the conversion of sulfur to long-chain lithium polysulfides. A cathodic peak, associated with a change in short-chain lithium polysulfides (Li_2_S_2_, Li_2_S), is observed at 2.04 V. Two anodic peaks appear at 2.36 and 2.42 V, which are associated with the recovery of sulfur from the lithium polysulfides [35,36]. Similarly, in the case of the CNT interlayer (Figure 4b), cathodic peaks appear at ~2.2 and ~1.95 V, and the reverse reactions occur at ~2.38 and ~2.46 V during the anodic scan. The cathodic peaks of the first and second CV curves differ slightly owing to the sulfur changing from alpha to beta phase [18,37]. The slightly higher voltage of the redox peaks for the cell containing the WNW/CNT interlayer suggests that the thiosulfate mediator on WNW enhances the low-order lithium polysulfide conversion kinetics [29,38]. In contrast, the CV curves of the cell without an interlayer show poor electrochemical activity, compared to the cells with interlayers (Figure 4c).

**Figure 5:**
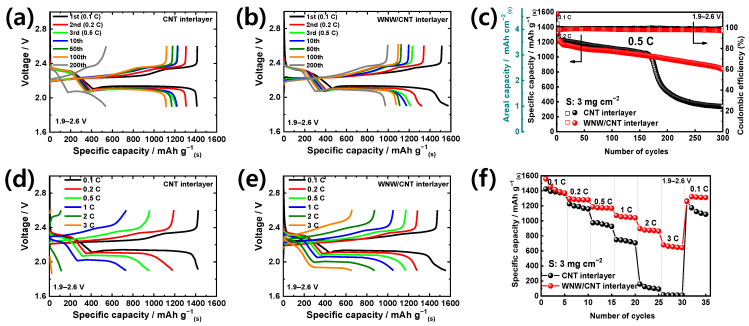
Voltage profiles of the lithium-sulfur cells, at 0.5 C, containing (**a**) CNT interlayer and (**b**) WNW/CNT interlayer, respectively. (**c**) Cycling performance of the lithium-sulfur cells. Voltage profiles of the lithium-sulfur cells, at different current densities, containing a (**d**) CNT interlayer and a (**e**) WNW/CNT interlayer, respectively. (**f**) Rate capability of the lithium-sulfur cells. The sulfur loading of all cells is 3 mg cm^−2^.

Figure 5 compares the effect of the WNW/CNT interlayer on the long-term cell capacity at different currents. As anticipated in the CV evaluation (Figure 4), a disappointing performance is observed for the sulfur cell without an interlayer (Figure S2). With the CNT interlayer (S-CNT cell), the lithium-sulfur cell delivers the highest capacity of 1224 mAh g^−1^ with a capacity fading rate of 0.09% per cycle for the first 160 cycles. After 160 cycles, the cell capacity decreases drastically until, at the 300th cycle, only 27.6% of the initial capacity is retained (Figure 5a,c). For the lithium-sulfur cell with the WNW/CNT interlayer (S-WNW/CNT cell), the delivered initial capacity of 1225 mAh g^−1^ fades at a rate of 0.11% per cycle (Figure 5b,c). Notably, ~68.3% (836.6 mAh g^−1^) of the initial capacity is retained after 300 cycles at 0.5 C, which is ~40% higher than the retained capacity of the S-CNT cell. After 300 cycles, the WNW/CNT cell demonstrates an areal capacity exceeding 2.5 mAh cm^−2^; moreover, it maintains a Coulombic efficiency (CE) of 97% for the duration of the cycling (Figure 5c).

The efficacy of the WNW/CNT interlayer is further evaluated in terms of rate capability. The S-CNT cell shows discharge capacities of 1424.6 mAh g^−1^ at 0.1 C and 734.7 mAh g^−1^ at 1 C, while the capacity is limited at 3 C (Figure 5d,f). When the current returns to a rate of 0.1 C, the capacity increases to 1263 mAh g^−1^; however, the cell is not able to maintain this capacity (Figure 5f). In contrast, the S-WNW/CNT cell can deliver a capacity of 656.0 mAh g^−1^ at a rate of 3 C (Figure 5e) and shows stable cycling performance after subsequent recovery of the current to 0.1 C (1316.2 mAh g^−1^). Environmental tests employing symmetric cells with Li_2_S_8_ electrolytes also demonstrate better electrochemical reversibility at high rates (Figure S3). The S-WNW/CNT cell shows a higher redox current density than the S-CNT cell at increasing scan rates, which verifies that the redox kinetics are significantly improved in the presence of liquid-phase polysulfides.

**Figure 6:**
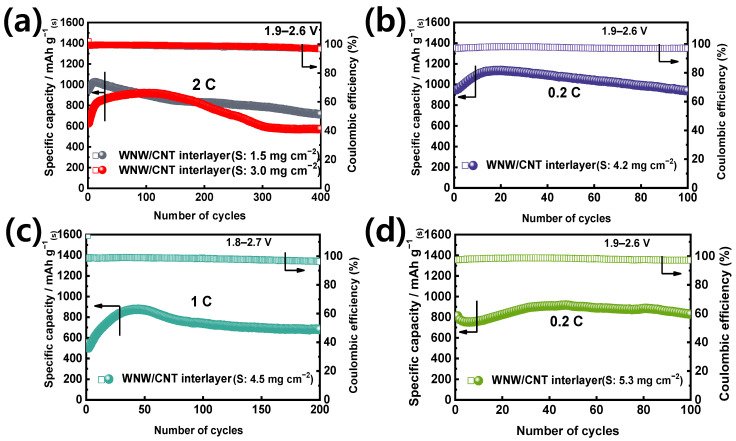
(**a**) Long-term cycling performance of lithium-sulfur cells with a WNW/CNT interlayer at 2 C (sulfur mass loading: 1.5 and 3.0 mg cm^−2^, respectively). Cycling performance of lithium-sulfur cells with sulfur mass loadings of (**b**) 4.2 mg cm^−2^ (0.2 C), (**c**) 4.5 mg cm^−2^ (1 C), and (**d**) 5.3 mg cm^−2^ (0.2 C).

The high-rate cycling performance of S-WNW/CNT cells was further tested at a rate of 2 C. When the mass loading of the active material is increased from 1.5 to 3.0 mg cm^−2^, the cell delivers a discharge capacity of 572 mAh g^−1^ after 400 cycles (Figure 6a).

The presence of the WNW/CNT interlayer enables the delivery of capacity even at increased sulfur loading densities of 4.2, 4.5, and 5.3 mg cm^−2^ (Figure 6b–d). It is worth highlighting that these superior cell performances at high sulfur loadings are achievable in the presence of WNW/CNT interlayers and may be associated with their electrochemical activity. Therefore, we conducted electrochemical tests on WNW/CNT and CNT interlayers without sulfur electrodes (Figure S4). The WNW/CNT interlayer has a discharge at the first cycle (127.8 mAh g^−1^), whereas the capacity during subsequent cycles is negligible. Evidently, the WNW/CNT interlayer does not participate in the electrochemical reaction of the sulfur electrode but facilitates electron transfer during cycling.

**Figure 7:**
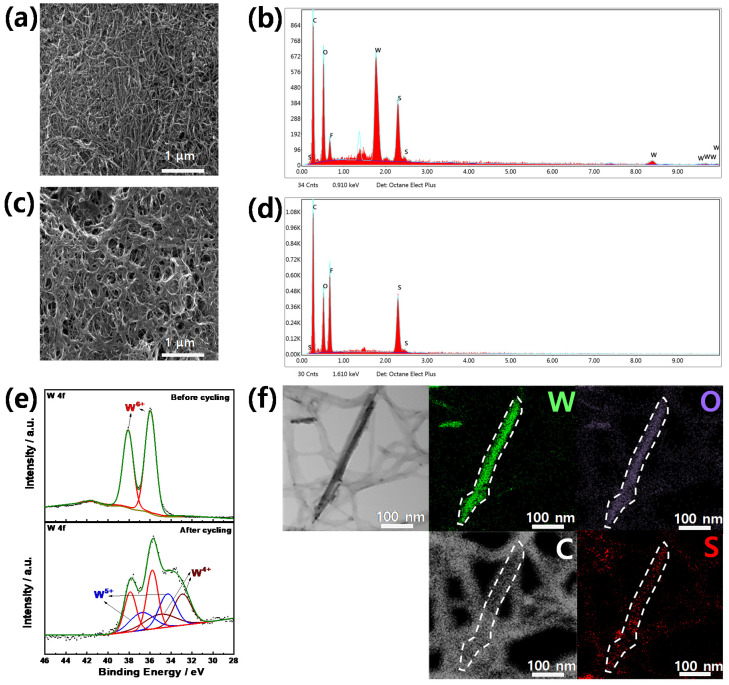
SEM images and area EDS measurements of WNW/CNT and CNT interlayers and their charged states after 100 cycles: (**a**,**b**) WNW/CNT interlayer and (**c**,**d**) CNT interlayer. (**e**) XPS spectra (W 4f) of the WNW/CNT interlayer before and after cycling. (**f**) TEM images of a cycled WNW/CNT interlayer with EDS mapping images of tungsten, oxygen, sulfur, and carbon.

To gain insight into the superior electrode performance of S-WNW/CNT cells, we observed the interlayers with charged states after extensive cycling (Figure 7). Compared to the morphology of the as-fabricated WNW/CNT interlayer (Figure 1d), the morphology of the WNW/CNT interlayer (Figure 7a,b) after cycling appears unchanged. However, the morphology of the CNT interlayer (Figure 7c,d) after cycling does differ from that of the as-fabricated CNT layer (Figure S1a), which could be attributed to the localized accumulation of lithium polysulfides since carbon has a low affinity for lithium polysulfides [23]. This heterogeneity of the CNT interlayer is likely to impede the facile conversion of sulfur to lithium polysulfides during long-term cycling, illustrated by the undesired capacity fading observed for the S-CNT cell; see Figure 5c. The WNW/CNT interlayer is capable of chemically adsorbing lithium polysulfides and promoting the redox reaction of lithium polysulfide owing to the catalytic effect of WNW; this suppresses the accumulation of lithium polysulfides on the interlayer.

**Figure 8:**
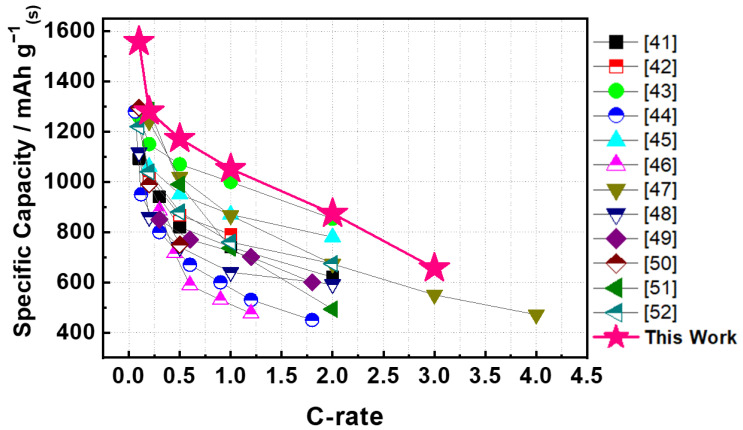
Comparison of the rate capabilities of lithium-sulfur batteries incorporating different types of interlayer or functional separators [41,42,43,44,45,46,47,48,49,50,51,52].

Although cycling does not alter the morphology of the WNW/CNT interlayer, the XPS results indicate a change in the chemical state of the interlayer (Figure 7e). After cycling, the binding energies of the two peaks associated with W^6+^ are ~0.2 eV less than those of the corresponding peaks before cycling, while additional peaks appear at 32.9, 34.3, 35.0, and 36.8 eV. The new peaks are associated with W^5+^ (34.3 and 36.8 eV) and W^4+^ (32.9 and 35.0 eV) [39], which form when WO_3_ is reduced during charge transfers between lithium polysulfide and WNW. The TEM-EDS mapping images support this relationship (Figure 7f). The homogeneous elemental distribution on the WNW results from the adherence of lithium polysulfide via thiosulfate mediation, which occurs more actively on the WNW surface than on that of the CNT, which has a weak affinity for lithium polysulfide [36,40]. These results are consistent with the interaction between the thiosulfate mediator and lithium polysulfide on WNW, as shown in Figure 2a, confirming that WO_3_ and polysulfides can be chemically bonded. Therefore, the WNW plays a pivotal role in capturing lithium polysulfide during electrochemical reactions and promoting the redox reaction of lithium polysulfide through catalytic action, thereby enhancing the electrochemical performance of sulfur electrodes in lithium-sulfur cells. The electrochemical performance of lithium-sulfur batteries incorporating a WNW/CNT interlayer is compared with that of lithium-sulfur batteries incorporating other types of interlayer or functional separators in Figure 8 and Table S1, highlighting the superiority of the present WNW/CNT interlayer.

## 3. Materials and Methods

### 3.1. Synthesis of WNW

WNWs were synthesized using a hydrothermal method [53]. Sodium tungstate dihydrate powder (3.29 g, Sigma Aldrich, St. Louis, MO, USA) was dissolved in deionized water (76 mL), and an aqueous HCl solution (3 M) was used to adjust the pH to 1.0. Ammonium sulfate (Sigma Aldrich, 2.64 g) was subsequently added to the solution to control the morphology of the WO_3_ product and stirred for 1 h. The solution was transferred into a Teflon-lined, stainless-steel autoclave (capacity: 100 mL) and heated at 180 °C for 24 h. After the reaction, the autoclave was cooled to room temperature. The WO_3_ precipitate, with a bright emerald-green color, was collected through filtration and washed with distilled water. To remove reactant residues, the product was rinsed with ethanol three times. Finally, the product was dried in a vacuum oven at 60 °C for 12 h.

### 3.2. Preparation of CNT Paper and WNW/CNT Paper

To produce CNT paper, multi-walled carbon nanotubes (MWCNTs, Hanwha Chem., Seoul, Korea, 100 mg) were dispersed in deionized water (200 mL) and isopropyl alcohol (8 mL) through ultrasonication for 10 min. The MWCNTs were retrieved through vacuum filtration using membrane paper (Advantec, Tokyo, Japan, No.2) and dried in an oven at 60 °C for 12 h. To fabricate WNW/CNT paper, WNWs (50 mg) and MWCNTs (50 mg) were mixed with a mixer-mill (Retsch, Haan, Germany, MM 400) at a frequency of 5 Hz for 15 min. The WNW/CNT paper was subsequently prepared following a process that is similar to making CNT paper.

After drying, the WNW/CNT paper was peeled from the membrane and cut into 16 mm-diameter circular disks for electrochemical testing. The weight of the interlayer was adjusted for each sulfur mass loading to achieve the required sulfur content ratio. If the sulfur mass loading was 3.0 mg cm^−2^, the weight per area of the interlayer was approximately 2.0 mg cm^−2^ (the overall sulfur content was ~48%).

### 3.3. Preparation of Electrodes

A sulfur cathode, for use with an interlayer, was prepared by applying a slurry, consisting of sulfur powder (Sigma Aldrich, 70 wt.%), carbon black Super-P (20 wt.%), and polyacrylic acid (PAA, 10 wt.%) in ethanol, onto carbon-coated aluminum foil, and then drying it overnight at 60 °C in an oven. A sulfur cathode, for use without an interlayer, was made of 48 wt.% sulfur, 32 wt.% Super-P, and 20 wt.% PAA binder. A WNW cathode, used to evaluate the electrochemical reactivity of WNW, was prepared in a similar way to the above sulfur cathodes. A slurry containing 80 wt.% WNW, 10 wt.% Super-P, and 10 wt.% PAA was applied to the gas diffusion layer (GDL).

### 3.4. Material Characterization

The morphology of the as-synthesized WNW and interlayers was investigated using scanning electron microscopy (SEM; Nova NanoSEM 450, FEI, Hillsboro, OR, USA) and transmission electron microscopy (TEM; JEM-2100F, JEOL, Tokyo, Japan). The crystal structures of the obtained products were characterized by X-ray diffractometry (MiniFlex600, Rigaku, Japan) using Cu Kα radiation. The surface area and pore size distribution of the WNWs were measured using a Quantachrome Autosorb iQ MP automated gas adsorption system with liquid nitrogen (at 77 K). X-ray photoelectron spectroscopy (XPS; Thermo Fisher Scientific, Waltham, MA, USA) was used to investigate the surface and chemical states of the WNWs; the binding energy values were calibrated based on the C 1s peak at 284.5 eV.

### 3.5. Electrochemical Measurements

Electrochemical tests were performed with coin-type cells (R2032) constructed with sulfur electrodes. WNW/CNT or CNT interlayers were sandwiched between the cathode and a microporous polypropylene film membrane (Celgard 2400) separator; the assembly was countered with a Li-foil anode. The prepared sulfur cathodes were cut into circular disks with diameters of 10 mm, and the sulfur mass loading levels on the electrodes ranged from ~1.5 to ~5.3 mg cm^−2^. The overall sulfur content in the electrodes was ~48%. The electrolyte solution contained 1 M lithium bis(trifluoromethanesulfonyl)imide (LiTFSI) and 0.4 M lithium nitrate (LiNO_3_) in a 1:1 (*v/v*) mixture of 1,3-dioxolane (DOL) and 1,2-dimethoxyethane (DME). The electrolyte/sulfur ratio (E/S ratio) was 20:1. All cells were assembled in an Ar-filled glove box (MBRAUN). Galvanostatic charge-discharge tests were conducted in constant current mode from 0.1 (167.5 mA g^−1^) to 3 C (5.025 A g^−1^) within a voltage range of 1.9 to 2.6 V at 30 °C, monitored using a battery testing system (TOSCAT-3100, Toyo System Co., Iwaki, Japan). For sulfur electrodes with high mass loading (4.5 mg cm^−2^), the cells were tested at 1 C currents in a voltage range of 1.8 to 2.7 V. Cyclic voltammetry (CV) measurements were carried out on a multi-channel potentiostat (VMP-3, Biologic) across the voltage range of 1.9 to 2.6 V at a scan rate of 0.1 mV s^−1^. The CV of the WNW cathode was tested at 0.05 mV s^−1^ using 1 M LiTFSI in a 1:1 (*v/v*) mixture of DOL and DME. Symmetric cells were assembled into an R2032 coin-type cell with a polypropylene separator, two identical electrodes (WNW/CNT interlayer, or CNT interlayer), and Li_2_S_8_ electrolyte (20.0 mL containing 0.25 M Li_2_S_8_ and 1 M LiTFSI in a 1:1 (*v/v*) mixture of DOL/DME). CV measurements of the symmetrical cells were conducted at different scan rates (−0.8 to 0.8 V).

## 4. Conclusions

We introduced a 1D-structured WNW/CNT interlayer into a lithium-sulfur battery to capture lithium polysulfides. As intended by the design, the presence of the WNW/CNT interlayer improves the electrode performance in terms of cyclability and rate capability even when the loading density of the active material is increased. The interlayer structure remains intact even after long-term cycling. We believe that this simply made WNW/CNT interlayer presents a promising approach to improve the performance of lithium-sulfur batteries. Its excellent performance is due to the trapping of lithium polysulfide in the interlayer by chemical adsorption and catalytic effect of the WNW as thiosulfate mediators.

## Data Availability

The data presented in this study are available on request from the corresponding author.

## Supplementary Materials

The following are available online. Figure S1: (a) SEM images of CNT interlayer with EDS mapping images of carbon, tungsten, oxygen. (b) Isotherm profiles and (c) pore size distributions of WNW. Figure S2: (a) Cycling performance and (b) voltage profiles of a lithium-sulfur cell without an interlayer. Figure S3: Cyclic voltammetry curves of the lithium-sulfur cells with a (a) WNW/CNT interlayer and (b) CNT interlayer, respectively, at different scan rates. Figure S4: (a) Cycling performances of the interlayers without sulfur electrodes. (b) Cyclic voltammetry (CV) profiles of WNW (1.5–3.8 V). Table S1: Comparison of overall electrochemical performances previously reported lithium-sulfur batteries incorporating different types of interlayer or functional separators.

## Sample Availability

Samples of the compounds are available from the authors.

## References

[1] Xue W., Shi Z., Suo L., Wang C., Wang Z., Wang H., So K.P., Maurano A., Yu D., Chen Y. (2019). Intercalation-conversion hybrid cathodes enabling Li–S full-cell architectures with jointly superior gravimetric and volumetric energy densities. Nat. Energy.

[2] Bruce P.G., Freunberger S.A., Hardwick L.J., Tarascon J.M. (2012). Li-O_2_ and Li-S batteries with high energy storage. Nat. Mater..

[3] Ji X., Lee K.T., Nazar L.F. (2009). A highly ordered nanostructured carbon-sulphur cathode for lithium-sulphur batteries. Nat. Mater..

[4] Hwang J.-Y., Myung S.-T., Sun Y.-K. (2017). Sodium-ion batteries: Present and future. Chem. Soc. Rev..

[5] Lu J., Li L., Park J.-B., Sun Y.-K., Wu F., Amine K. (2014). Aprotic and Aqueous Li–O_2_ Batteries. Chem. Rev..

[6] Liu D., Zhang C., Zhou G., Lv W., Ling G., Zhi L., Yang Q.H. (2018). Catalytic Effects in Lithium–Sulfur Batteries: Promoted Sulfur Transformation and Reduced Shuttle Effect. Adv. Sci..

[7] Kumar R., Liu J., Hwang J.-Y., Sun Y.-K. (2018). Recent research trends in Li–S batteries. J. Mater. Chem. A.

[8] Wang H.Q., Zhang W.C., Xu J.Z., Guo Z.P. (2018). Advances in Polar Materials for Lithium-Sulfur Batteries. Adv. Funct. Mater..

[9] Huang S., Lim Y.V., Zhang X., Wang Y., Zheng Y., Kong D., Ding M., Yang S.A., Yang H.Y. (2018). Regulating the polysulfide redox conversion by iron phosphide nanocrystals for high-rate and ultrastable lithium-sulfur battery. Nano Energy.

[10] Wu P., Chen L.H., Xiao S.S., Yu S., Wang Z., Li Y., Su B.L. (2018). Insight into the positive effect of porous hierarchy in S/C cathodes on the electrochemical performance of Li-S batteries. Nanoscale.

[11] Manthiram A., Fu Y., Chung S.H., Zu C., Su Y.S. (2014). Rechargeable lithium-sulfur batteries. Chem. Rev..

[12] Mikhaylik Y.V., Akridge J.R. (2004). Polysulfide shuttle study in the Li/S battery system. J. Electrochem. Soc..

[13] Chung S.-H., Chang C.-H., Manthiram A. (2018). Progress on the Critical Parameters for Lithium-Sulfur Batteries to be Practically Viable. Adv. Funct. Mater..

[14] Mikhaylik Y.V., Kovalev I., Schock R., Kumaresan K., Xu J., Affinito J. (2010). High Energy Rechargeable Li-S Cells for EV Application: Status, Remaining Problems and Solutions. ECS Trans..

[15] Zhang Y., Gao Z., Song N., He J., Li X. (2018). Graphene and its derivatives in lithium–sulfur batteries. Mater. Today Energy.

[16] Jeong Y.C., Kim J.H., Nam S., Park C.R., Yang S.J. (2018). Rational Design of Nanostructured Functional Interlayer/Separator for Advanced Li-S Batteries. Adv. Funct. Mater..

[17] Huang J.-Q., Zhang Q., Wei F. (2015). Multi-functional separator/interlayer system for high-stable lithium-sulfur batteries: Progress and prospects. Energy Storage Mater..

[18] Luo L., Qin X., Wu J., Liang G., Li Q., Liu M., Kang F., Chen G., Li B. (2018). An interwoven MoO_3_@CNT scaffold interlayer for high-performance lithium–sulfur batteries. J. Mater. Chem. A.

[19] Yue X.-Y., Li X.-L., Meng J.-K., Wu X.-J., Zhou Y.-N. (2018). Padding molybdenum net with Graphite/MoO_3_ composite as a multi-functional interlayer enabling high-performance lithium-sulfur batteries. J. Power Sources.

[20] Xiao Z., Yang Z., Wang L., Nie H., Zhong M., Lai Q., Xu X., Zhang L., Huang S. (2015). A Lightweight TiO_2_/Graphene Interlayer, Applied as a Highly Effective Polysulfide Absorbent for Fast, Long-Life Lithium-Sulfur Batteries. Adv. Mater..

[21] Dong W., Meng L., Hong X., Liu S., Shen D., Xia Y., Yang S. (2020). MnO_2_/rGO/CNTs Framework as a Sulfur Host for High-Performance Li-S Batteries. Molecules.

[22] Zhao T., Ye Y., Peng X., Divitini G., Kim H.-K., Lao C.-Y., Coxon P.R., Xi K., Liu Y., Ducati C. (2016). Advanced Lithium-Sulfur Batteries Enabled by a Bio-Inspired Polysulfide Adsorptive Brush. Adv. Funct. Mater..

[23] Yang L., Li G., Jiang X., Zhang T., Lin H., Lee J.Y. (2017). Balancing the chemisorption and charge transport properties of the interlayer in lithium–sulfur batteries. J. Mater. Chem. A.

[24] Liu F., Xiao Q., Wu H.B., Sun F., Liu X., Li F., Le Z., Shen L., Wang G., Cai M. (2017). Regenerative Polysulfide-Scavenging Layers Enabling Lithium-Sulfur Batteries with High Energy Density and Prolonged Cycling Life. ACS Nano.

[25] Lin H.B., Zhang S.L., Zhang T.R., Ye H.L., Yao Q.F., Zheng G.W., Lee J.Y. (2018). Elucidating the Catalytic Activity of Oxygen Deficiency in the Polysulfide Conversion Reactions of Lithium-Sulfur Batteries. Adv. Energy Mater..

[26] Choi S., Seo D.H., Kaiser M.R., Zhang C., van der Laan T., Han Z.J., Bendavid A., Guo X., Yick S., Murdock A.T. (2019). WO_3_ nanolayer coated 3D-graphene/sulfur composites for high performance lithium/sulfur batteries. J. Mater. Chem. A.

[27] Yang X., Zu H., Luo L., Zhang H., Li J., Yi X., Liu H., Wang F., Song J. (2020). Synergistic tungsten oxide/N, S co-doped carbon nanofibers interlayer as anchor of polysulfides for high-performance lithium-sulfur batteries. J. Alloys Compd..

[28] Zhang J., Tu J.-P., Xia X.-H., Wang X.-L., Gu C.-D. (2011). Hydrothermally synthesized WO_3_ nanowire arrays with highly improved electrochromic performance. J. Mater. Chem..

[29] Liang X., Hart C., Pang Q., Garsuch A., Weiss T., Nazar L.F. (2015). A highly efficient polysulfide mediator for lithium-sulfur batteries. Nat. Commun..

[30] Naumkin A.V., Kraut-Vass A., Gaarenstroom S.W., Powell C.J. (2012). NIST Standard Reference Database 20, Version 4.1.

[31] Kondalkar V.V., Mali S.S., Kharade R.R., Khot K.V., Patil P.B., Mane R.M., Choudhury S., Patil P.S., Hong C.K., Kim J.H. (2015). High performing smart electrochromic device based on honeycomb nanostructured h-WO_3_ thin films: Hydrothermal assisted synthesis. Dalton Trans..

[32] Hur Y.G., Lee D.-W., Lee K.-Y. (2016). Hydrocracking of vacuum residue using NiWS_(x)_ dispersed catalysts. Fuel.

[33] Ishutenko D., Minaev P., Anashkin Y., Nikulshina M., Mozhaev A., Maslakov K., Nikulshin P. (2017). Potassium effect in K-Ni(Co)PW/Al_2_O_3_ catalysts for selective hydrotreating of model FCC gasoline. Appl. Catal. B.

[34] Nikulshina M., Mozhaev A., Lancelot C., Marinova M., Blanchard P., Payen E., Lamonier C., Nikulshin P. (2018). MoW synergetic effect supported by HAADF for alumina based catalysts prepared from mixed SiMonW12-n heteropolyacids. Appl. Catal. B.

[35] Hwang J.-Y., Kim H.M., Lee S.-K., Lee J.-H., Abouimrane A., Khaleel M.A., Belharouak I., Manthiram A., Sun Y.-K. (2016). High-Energy, High-Rate, Lithium-Sulfur Batteries: Synergetic Effect of Hollow TiO_2_-Webbed Carbon Nanotubes and a Dual Functional Carbon-Paper Interlayer. Adv. Energy Mater..

[36] Hwang J.-Y., Kim H.M., Shin S., Sun Y.-K. (2018). Designing a High-Performance Lithium-Sulfur Batteries Based on Layered Double Hydroxides-Carbon Nanotubes Composite Cathode and a Dual-Functional Graphene-Polypropylene-Al_2_O_3_ Separator. Adv. Funct. Mater..

[37] Walus S., Barchasz C., Colin J.F., Martin J.F., Elkaim E., Lepretre J.C., Alloin F. (2013). New insight into the working mechanism of lithium-sulfur batteries: In situ and operando X-ray diffraction characterization. Chem. Commun..

[38] Liang X., Kwok C.Y., Lodi-Marzano F., Pang Q., Cuisinier M., Huang H., Hart C.J., Houtarde D., Kaup K., Sommer H. (2016). Tuning Transition Metal Oxide-Sulfur Interactions for Long Life Lithium Sulfur Batteries: The “Goldilocks” Principle. Adv. Energy Mater..

[39] Xie F.Y., Gong L., Liu X., Tao Y.T., Zhang W.H., Chen S.H., Meng H., Chen J. (2012). XPS studies on surface reduction of tungsten oxide nanowire film by Ar^+^ bombardment. J. Electron Spectrosc. Relat. Phenom..

[40] Pang Q., Kundu D., Cuisinier M., Nazar L.F. (2014). Surface-enhanced redox chemistry of polysulphides on a metallic and polar host for lithium-sulphur batteries. Nat. Commun..

[41] Liang G., Wu J., Qin X., Liu M., Li Q., He Y.B., Kim J.K., Li B., Kang F. (2016). Ultrafine TiO_2_ Decorated Carbon Nanofibers as Multifunctional Interlayer for High-Performance Lithium-Sulfur Battery. ACS Appl. Mater. Interfaces.

[42] Chang C.-H., Chung S.-H., Manthiram A. (2015). Ultra-lightweight PANiNF/MWCNT-functionalized separators with synergistic suppression of polysulfide migration for Li–S batteries with pure sulfur cathodes. J. Mater. Chem. A.

[43] Lee C.L., Kim I.D. (2015). A hierarchical carbon nanotube-loaded glass-filter composite paper interlayer with outstanding electrolyte uptake properties for high-performance lithium-sulphur batteries. Nanoscale.

[44] Kim H.M., Hwang J.Y., Manthiram A., Sun Y.-K. (2016). High-Performance Lithium-Sulfur Batteries with a Self-Assembled Multiwall Carbon Nanotube Interlayer and a Robust Electrode-Electrolyte Interface. ACS Appl. Mater. Interfaces.

[45] Song R., Fang R., Wen L., Shi Y., Wang S., Li F. (2016). A trilayer separator with dual function for high performance lithium–sulfur batteries. J. Power Sources.

[46] Lin C., Zhang W., Wang L., Wang Z., Zhao W., Duan W., Zhao Z., Liu B., Jin J. (2016). A few-layered Ti_3_C_2_ nanosheet/glass fiber composite separator as a lithium polysulphide reservoir for high-performance lithium–sulfur batteries. J. Mater. Chem. A.

[47] Lu Y., Gu S., Guo J., Rui K., Chen C., Zhang S., Jin J., Yang J., Wen Z. (2017). Sulfonic Groups Originated Dual-Functional Interlayer for High Performance Lithium-Sulfur Battery. ACS Appl. Mater. Interfaces.

[48] Kim J.H., Seo J., Choi J., Shin D., Carter M., Jeon Y., Wang C., Hu L., Paik U. (2016). Synergistic Ultrathin Functional Polymer-Coated Carbon Nanotube Interlayer for High Performance Lithium-Sulfur Batteries. ACS Appl. Mater. Interfaces.

[49] Guo P., Liu D., Liu Z., Shang X., Liu Q., He D. (2017). Dual functional MoS_2_/graphene interlayer as an efficient polysulfide barrier for advanced lithium-sulfur batteries. Electrochim. Acta.

[50] Chang C.-H., Chung S.-H., Manthiram A. (2017). Transforming waste newspapers into nitrogen-doped conducting interlayers for advanced Li–S batteries. Sustain. Energy Fuels.

[51] Shi H., Zhao X., Wu Z.-S., Dong Y., Lu P., Chen J., Ren W., Cheng H.-M., Bao X. (2019). Free-standing integrated cathode derived from 3D graphene/carbon nanotube aerogels serving as binder-free sulfur host and interlayer for ultrahigh volumetric-energy-density lithium sulfur batteries. Nano Energy.

[52] Zheng B., Yu L., Li N., Xi J. (2020). Efficiently immobilizing and converting polysulfide by a phosphorus doped carbon microtube textile interlayer for high-performance lithium-sulfur batteries. Electrochim. Acta.

[53] Wang H.P., Ma G.F., Tong Y.C., Yang Z.R. (2018). Biomass carbon/polyaniline composite and WO_3_ nanowire-based asymmetric supercapacitor with superior performance. Ionics.

