# Supplementary material for: WO3 Nanowire/Carbon Nanotube Interlayer as a Chemical Adsorption Mediator for High-Performance Lithium-Sulfur Batteries
Source: Molecules. 2021 Jan 13;26(2):377. doi: 10.3390/molecules26020377 (PMC7828354; doi:10.3390/molecules26020377)
Supplement: Supplementary file 1 [file molecules-26-00377-s001.pdf]

WO_3 nanowire/carbon nanotube interlayer as a chemical adsorption mediator for high-performance lithium-sulfur batteries

Sang-Kyu Lee [1], Hun Kim [1], Sangin Bang [1], Seung-Taek Myung [2,*] and Yang-Kook Sun [1,*]

[1] Department of Energy Engineering, Hanyang University, Seoul 04763, South Korea; chemiphile7@gmail.com (S.-K.L.); rlagns0829@nate.com (H.K.); b930403@gmail.com (S.B.)

[2] Department of Nanotechnology and Advanced Materials Engineering & Sejong Battery Institute, Sejong University, Seoul 05006, South Korea;

* Correspondence: smyung@sejong.ac.kr (S.-T.M.) yksun@hanyang.ac.kr (Y.-K.S.); Tel.: +82-2-2220-0524

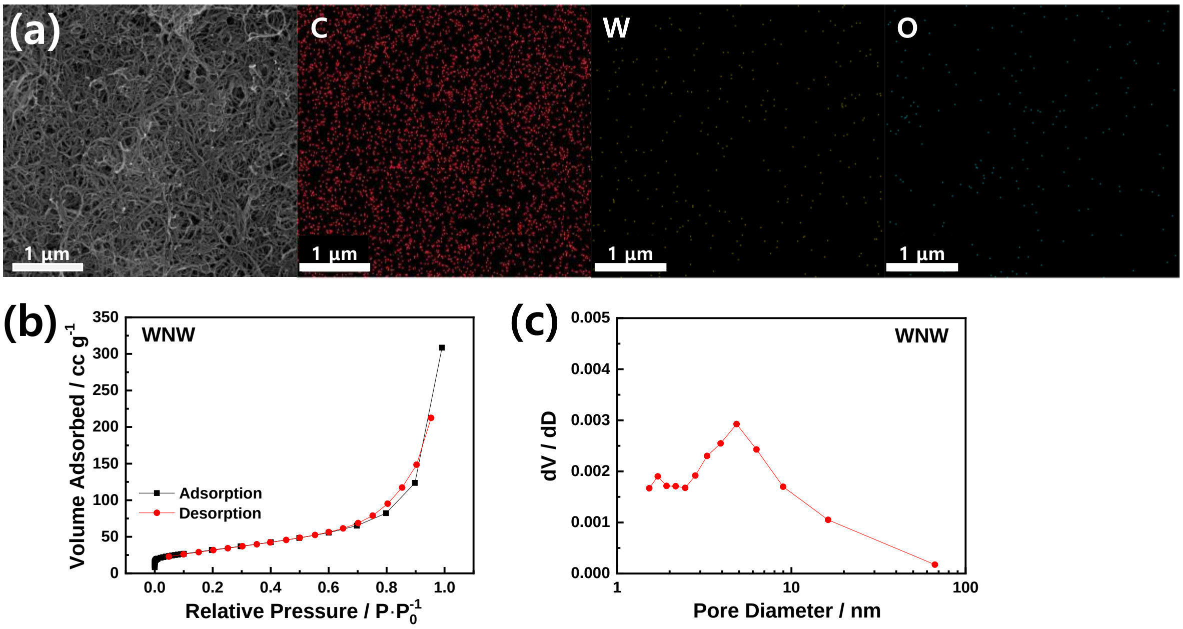

Figure S1. (a) SEM images of CNT interlayer with EDS mapping images of carbon, tungsten, oxygen. (b) Isotherm profiles and (c) pore size distributions of WNW.

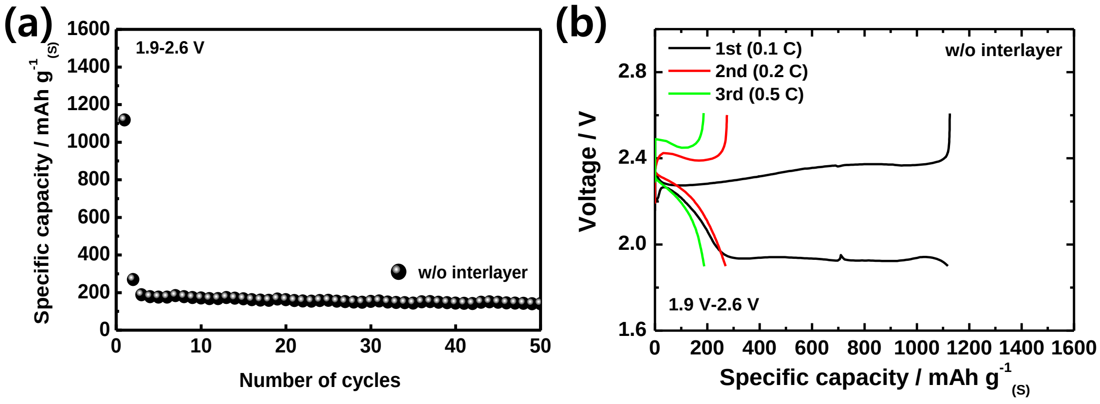

Figure S2. (a) Cycling performance and (b) voltage profiles of a lithium-sulfur cell without an interlayer.

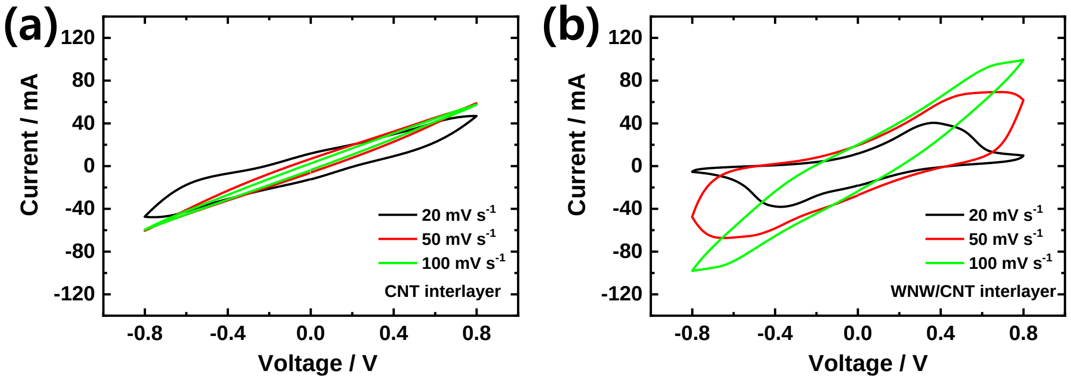

Figure S3. Cyclic voltammetry curves of the lithium-sulfur cells with a (a) WNW/CNT interlayer and (b) CNT interlayer, respectively, at different scan rates.

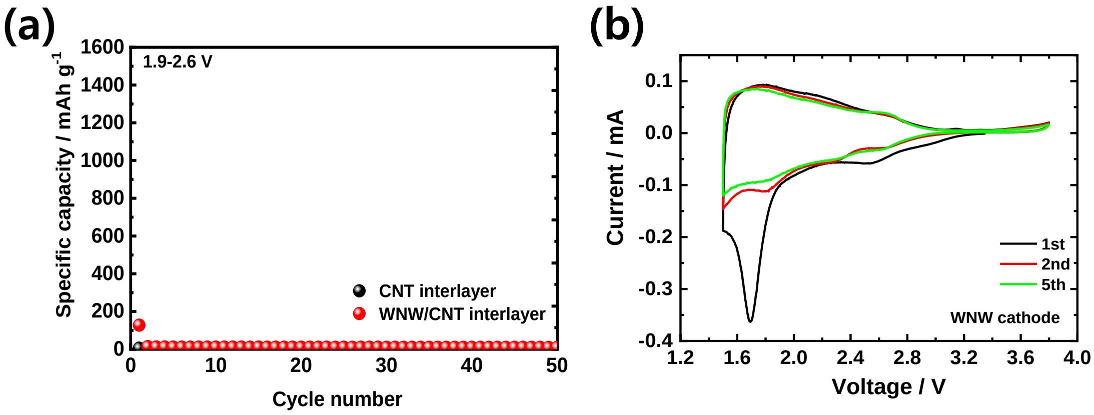

Figure S4. (a) Cycling performances of the interlayers without sulfur electrodes. (b) CV profiles of WNW (1.5–3.8 V).

Table S1 Comparison of overall electrochemical performances previously reported lithium-sulfur batteries incorporating different types of interlayer or functional separators.

Samples	Sulfur content in cathode (%)	Overall S content (%)	Sulfur loading (mg cm^{-2})	Specific capacity (mAh g^{-1})(s)	Cycle number	Capacity fading rate (%/cycle)	Rate capability	Reference
CNF-T interlayer	60	42.5	0.8	1328 (0.2 C)	500	0.121	1091 (0.1 C)<br>940 (0.3 C)<br>820 (0.5 C)<br>740 (1 C)<br>620 (2 C)	[S1]
PANiNF/CNT separator	60	59.7	1.4	1020 (0.2 C)	100	0.3	1020 (0.2 C)<br>867 (0.5 C)<br>791 (1 C)	[S2]
Glass fiber/CNT interlayer	70	56.6	1.6	1111.7 (0.2 C)	230	0.12	1260 (0.1 C)<br>1150 (0.2 C)<br>1070 (0.5 C)<br>1000 (1 C)	[S3]

							853 (2 C)	
Self-assembled CNT interlayer	80	52.6	3.0	851 (0.5 C)	100	0.042	1280 (0.06 C)<br>950 (0.12 C)<br>800 (0.3 C)<br>670 (0.6 C)<br>600 (0.9 C)<br>530 (1.2 C)<br>450 (1.8 C)	[S4]
Graphene/PP/Al_2O_3 separator	60	45.1	2.4	1068 (0.2 C)	100	0.25	1060 (0.2 C)<br>950 (0.5 C)<br>870 (1 C)<br>780 (2 C)	[S5]
Ti_3C_2 nanosheet/glass fiber separator	70	59.0	1.9	820 (0.3 C)	100	0.12	886 (0.3 C)<br>716 (0.45 C)<br>589 (0.6 C)<br>530 (0.9 C)<br>476 (1.2 C)	[S6]

SRGO/glass fiber interlayer	53.3	50.6-51.2	1.2-1.5	1320 (0.5 C)	250	0.18	1240 (0.2 C)<br>1019 (0.5 C)<br>866 (1 C)<br>673 (2 C)<br>550 (3 C)<br>471 (4 C)	[S7]
PAA-SWNT interlayer	65	54.3	2.7	770 (1 C)	200	0.13	1117 (0.1 C)<br>860 (0.2 C)<br>740 (0.5 C)<br>640 (1 C)<br>592 (2 C)	[S8]
MoS_2/graphene interlayer	60	43.6-48.0	0.8-1.2	840 (0.6 C)	200	0.13	850 (0.3 C)<br>770 (0.6 C)<br>701 (1.2 C)<br>600 (1.8 C)	[S9]
N-doped conductive interlayer	75	50.0	3.0	990 (0.2 C)	200	0.24	1295 (0.1 C)<br>990 (0.2 C)<br>750 (0.5 C)	[S10]

Graphene/CNT aerogels interlayer	50	48.0	2.46	1128 (0.5 C)	100	0.25	1286 (0.2 C)<br>990 (0.5 C)<br>736 (1 C)<br>493 (2 C)	[S11]
P-doped C microtube textile interlayer	60	36.3-37.2	2.9-3.1	788 (1 C)	100	0.07	1220 (0.1 C)<br>1040 (0.2 C)<br>880 (0.5 C)<br>760 (1 C)<br>674 (2 C)	[S12]
WNW/CNT interlayer	70	48.0	3.0	1225 (0.5 C)	300	0.11	1558 (0.1 C)<br>1281 (0.2 C)<br>1172 (0.5 C)<br>1052 (1 C)<br>874 (2 C)<br>656 (3 C)	This work

References

S1. Liang, G.; Wu, J.; Qin, X.; Liu, M.; Li, Q.; He, Y. B.; Kim, J. K.; Li, B.; Kang, F., Ultrafine TiO_2 Decorated Carbon Nanofibers as Multifunctional Interlayer for High-Performance Lithium-Sulfur Battery. *ACS Appl. Mater. Interfaces* **2016,** *8*, 23105-23113.

S2. Chang, C.-H.; Chung, S.-H.; Manthiram, A., Ultra-lightweight PANiNF/MWCNT-functionalized separators with synergistic suppression of polysulfide migration for Li–S batteries with pure sulfur cathodes. *J. Mater. Chem. A* **2015,** *3*, 18829-18834.

S3. Lee, C. L.; Kim, I. D., A hierarchical carbon nanotube-loaded glass-filter composite paper interlayer with outstanding electrolyte uptake properties for high-performance lithium-sulphur batteries. *Nanoscale* **2015,** *7*, 10362-10367.

S4. Kim, H. M.; Hwang, J. Y.; Manthiram, A.; Sun, Y.-K., High-Performance Lithium-Sulfur Batteries with a Self-Assembled Multiwall Carbon Nanotube Interlayer and a Robust Electrode-Electrolyte Interface. *ACS Appl. Mater. Interfaces* **2016,** *8*, 983-987.

S5. Song, R.; Fang, R.; Wen, L.; Shi, Y.; Wang, S.; Li, F., A trilayer separator with dual function for high performance lithium–sulfur batteries. *J. Power sources* **2016,** *301*, 179-186.

S6. Lin, C.; Zhang, W.; Wang, L.; Wang, Z.; Zhao, W.; Duan, W.; Zhao, Z.; Liu, B.; Jin, J., A few-layered Ti_3C_2 nanosheet/glass fiber composite separator as a lithium polysulphide reservoir for high-performance lithium–sulfur batteries. *J. Mater. Chem. A* **2016,** *4*, 5993-5998.

S7. Lu, Y.; Gu, S.; Guo, J.; Rui, K.; Chen, C.; Zhang, S.; Jin, J.; Yang, J.; Wen, Z., Sulfonic Groups Originated Dual-Functional Interlayer for High Performance Lithium-Sulfur Battery. *ACS Appl. Mater. Interfaces* **2017,** *9*, 14878-14888.

S8. Kim, J. H.; Seo, J.; Choi, J.; Shin, D.; Carter, M.; Jeon, Y.; Wang, C.; Hu, L.; Paik, U., Synergistic Ultrathin Functional Polymer-Coated Carbon Nanotube Interlayer for High Performance Lithium-Sulfur Batteries. *ACS Appl. Mater. Interfaces* **2016,** *8*, 20092-20099.

S9. Guo, P.; Liu, D.; Liu, Z.; Shang, X.; Liu, Q.; He, D., Dual functional MoS_2/graphene interlayer as an efficient polysulfide barrier for advanced lithium-sulfur batteries. *Electrochim. Acta* **2017,** *256*, 28-36.

S10. Chang, C.-H.; Chung, S.-H.; Manthiram, A., Transforming waste newspapers into nitrogen-doped conducting interlayers for advanced Li–S batteries. *Sustainable Energy Fuels* **2017,** *1*, 444-449.

S11. Shi, H.; Zhao, X.; Wu, Z.-S.; Dong, Y.; Lu, P.; Chen, J.; Ren, W.; Cheng, H.-M.; Bao, X., Free-standing integrated cathode derived from 3D graphene/carbon nanotube aerogels serving as binder-free sulfur host and interlayer for ultrahigh volumetric-energy-density lithium sulfur batteries. *Nano Energy* **2019,** *60*, 743-751.

S12. Zheng, B.; Yu, L.; Li, N.; Xi, J., Efficiently immobilizing and converting polysulfide by a phosphorus doped carbon microtube textile interlayer for high-performance lithium-sulfur batteries. *Electrochim. Acta* **2020,** *345*, 136186.
